# Supplementary material for: Acute kidney injury and adverse renal events in patients receiving SGLT2-inhibitors: A systematic review and meta-analysis
Source: PLoS Med. 2019 Dec 9;16(12):e1002983. doi: 10.1371/journal.pmed.1002983 (PMC6901179; doi:10.1371/journal.pmed.1002983)
Supplement: S2 Text — (DOCX) [file pmed.1002983.s002.docx]

**Analysis plan**

(15.02.2019)

[data-driven changes highlighted in red]

| - systematic review/ meta-analysis to investigate whether there is a difference in the risk of AKI in patients on long-term therapy with SGLT2 inhibitors (dapagliflotzin/ canagliflozin/ empagliflozin/ sotagliflozin/ ertugliflozin/ ipragliflozin/ luseogliflozin/ tofogliflozin) compared to patients not taking this medication |
| --- |
|  |
| - search strategy: PUBMED, Embase, Cochrane Database, Clinicaltrials.gov search using search terms |
|  |
| **(dapagliflozin OR canagliflozin OR empagliflozin OR sotagliflozin OR ertugliflozin OR ipragliflozin OR luseogliflozin OR tofogliflozin OR SGLT2 OR (sodium glucose transporter 2)) AND (acute kidney injury OR acute kidney damage OR acute kidney failure OR acute renal failure OR hypotension OR hypovolemia OR dehydration OR real world OR propensity OR observational OR randomized OR randomisation OR placebo OR Clinical Trial[ptyp])** |
|  |
| - inclusion criteria: studies in human subjects (RCTs/ observational studies/ case series) stating number of cases of outcome of interest developing in patients on SGLT2 inhibitors vs patients on (placebo/ other second-line oral antihyperglycemic agent) |
|  |
|  |
| - exclusion criteria:  animal studies, case reports, expert opinion, studies focusing on a different drug class  *AMENDED 28.03.2019:* duration <12 weeks  *AMENDED 07.04.2019:* 0 events for outcomes of interest |
|  |
| - outcomes of interest: |
| -- characteristics of study: study type, author, year, number of participants, inclusion and exclusion criteria of study, definition of intervention and control groups |
| -- baseline characteristics of study population: age/ sex/ HbA1c/ % coronary heart disease, average eGFR, % eGFR<30% |
| -- outcomes: |
| ---acute kidney injury |
| 1)      Analysis SAE-AKI (hospitalisation for AKI) *amended 30.03.19*  2)      Analysis AKI (non-serious and serious) *amended 30.03.19* |
| --- hypovolemia |
| --- other renal adverse events |
| - assessment for risk of bias: Cochrane risk of bias assessment  Data collection form see Fig. 1. |

|  | **Study Characteristics** | | | | | | | **Study definition** | | | |  | **SGLT2 inhibitor** | | | | | | | | | | | | | **PLACEBO OR COMPARATOR** | | | | | | | | | | | | | |
| --- | --- | --- | --- | --- | --- | --- | --- | --- | --- | --- | --- | --- | --- | --- | --- | --- | --- | --- | --- | --- | --- | --- | --- | --- | --- | --- | --- | --- | --- | --- | --- | --- | --- | --- | --- | --- | --- | --- | --- |
| **Nr.** | **Study (citation)** | **NCT number** | **RCT/ observ** | **Blinded (yes/ no)** | **background DM therapy** | **Data source of outcome** | **Study Duration** | **AKI (1) SAE** | **AKI (2)**  **AE** | **Other adverse renal event** | **Volume depletion** | **Drug** | **dose (mg)** | **patients (n)** | **age (y)** | **sex (% male)** | **eGFR (ml/min)** | **SBP (mmHg)** | **CVD (%)** | **CKD (%)** | **eGFR <60 (%)** | **AKI (1) (n)** | **AKI (2) (n)** | **Adverse renal event (n)** | **Volume depletion (n)** | **Drug** | **dose (mg)** | **patients (n)** | **age (y)** | **sex (% male)** | **eGFR (ml/min)** | **SBP (mmHg)** | **CVD (%)** | **CKD (%)** | **eGFR <60 (%)** | **AKI (1) (n)** | **AKI (2) (n)** | **Adverse renal event (n)** | **Volume depletion (n)** |
| 1 |  |  |  |  |  |  |  |  |  |  |  |  |  |  |  |  |  |  |  |  |  |  |  |  |  |  |  |  |  |  |  |  |  |  |  |  |  |  |  |
| 2 |  |  |  |  |  |  |  |  |  |  |  |  |  |  |  |  |  |  |  |  |  |  |  |  |  |  |  |  |  |  |  |  |  |  |  |  |  |  |  |
| 3 |  |  |  |  |  |  |  |  |  |  |  |  |  |  |  |  |  |  |  |  |  |  |  |  |  |  |  |  |  |  |  |  |  |  |  |  |  |  |  |
| 4 |  |  |  |  |  |  |  |  |  |  |  |  |  |  |  |  |  |  |  |  |  |  |  |  |  |  |  |  |  |  |  |  |  |  |  |  |  |  |  |

Fig 1: Data collection form

Analysis

- Software: Comprehensive Meta Analysis
- OR, 95% confidence intervals, I^2^, p-Values
  - AKI SAE in RCTs and cohort studies
  - AKI AE in RCTs and cohort studies
  - Other adverse renal events in RCTs (not in cohort studies)
  - Volume depletion in RCTs (not in cohort studies)
  - *AMENDED 02.04.2019*: AKI in cohorts with eGFR <60
- Presentation as Forest Plots
- *AMENDED 29.03.2019*: Meta-regression analysis did not show an influence of dose (low/ medium/ high) of SGLT2 inhibitor on risk of AKI (Fig 2, data not shown in main paper) 🡪 decision made to pool different SGLT2 doses into one data point for each study

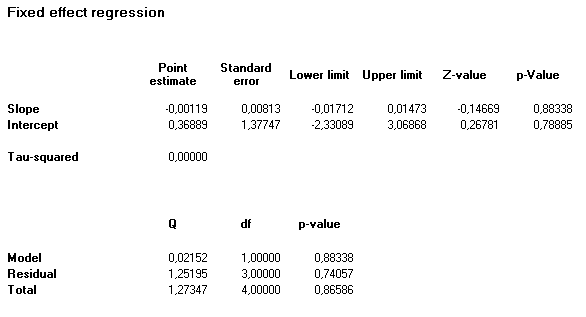


Fig 2: Metaregression of drug dose (low 1/ medium 2/ high 3) on log odds ratio for AKI

- *Amended 02.10.19* Further analyses in response to reviewer comments:
  - Subgroup analysis for effect of SGLT2 type on risk of combined adverse renal outcomes
  - subgroup analysis for effect of SGLT2 type on risk of volume depletion
  - meta-regression for effect of HbA1c, eGFR and BP on risk of combined adverse renal outcomes
