# Supplementary figures and images for: Acute kidney injury and adverse renal events in patients receiving SGLT2-inhibitors: A systematic review and meta-analysis
Source: PLoS Med. 2019 Dec 9;16(12):e1002983. doi: 10.1371/journal.pmed.1002983 (PMC6901179; doi:10.1371/journal.pmed.1002983)

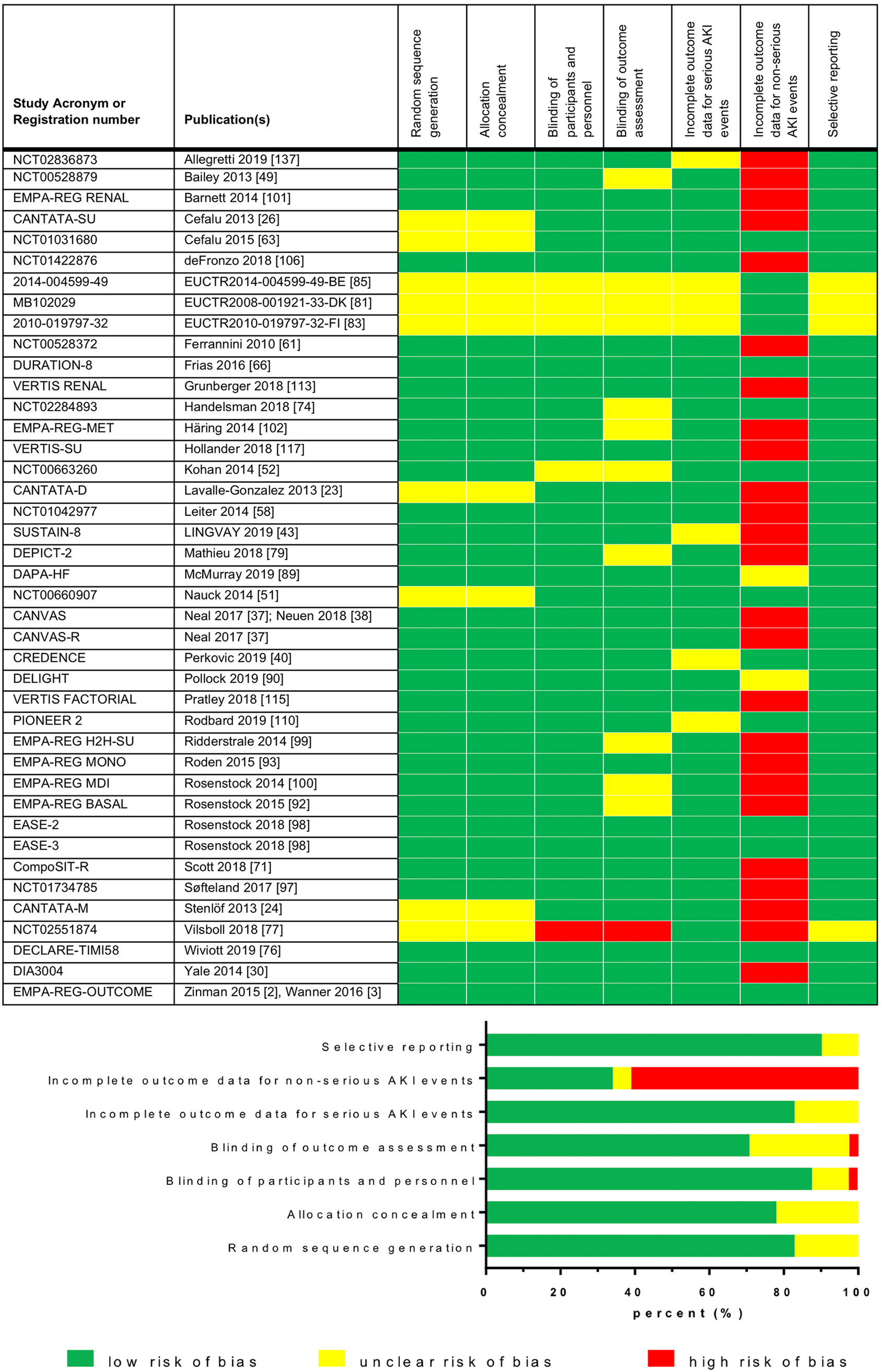

Supplement: S1 Fig — AE, adverse event; AKI, acute kidney injury; SAE, serious AE. (TIF) [file pmed.1002983.s007.tif]

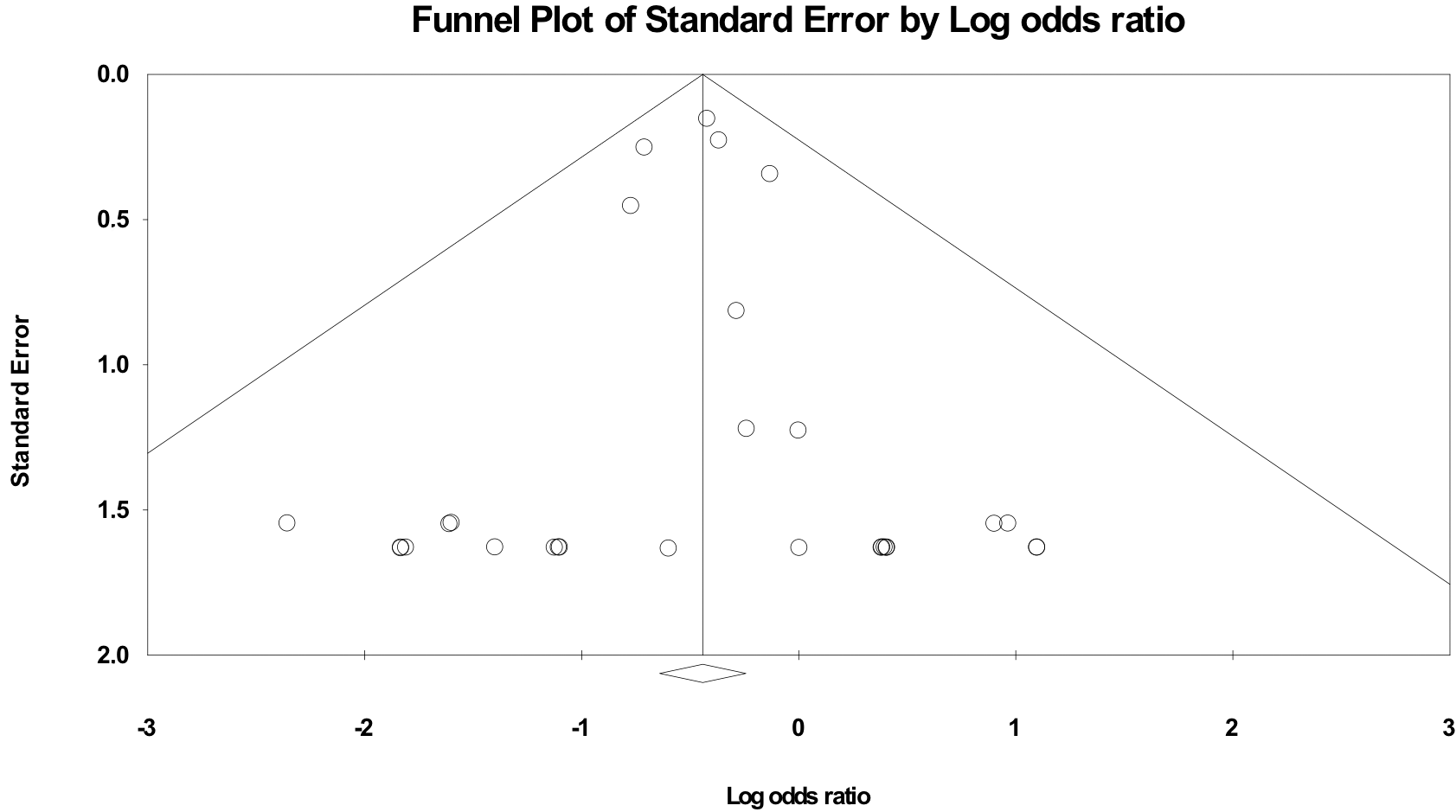

Supplement: S2 Fig — (TIF) [file pmed.1002983.s008.tif]

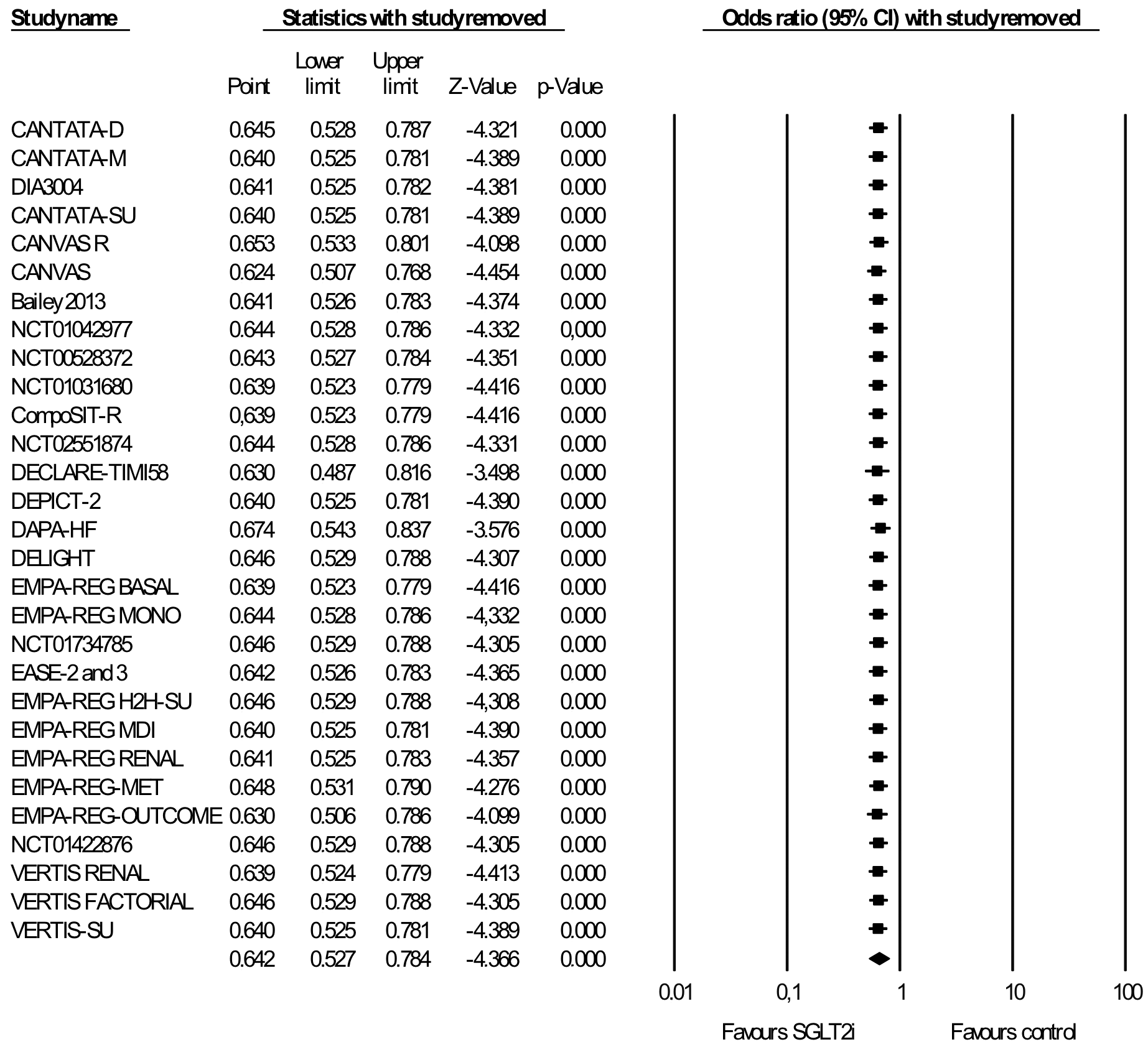

Supplement: S3 Fig — This analysis suggests that the overall result was not due to a single study. Even when the results of the largest study (DECLARE) was removed, the result remained highly significant (p < 0.001). AE, adverse event; AKI, acute kidney injury; SAE, serious AE; SGLT2i, sodium-glucose cotransporter-2 inhibitor. (TIF) [file pmed.1002983.s009.tif]

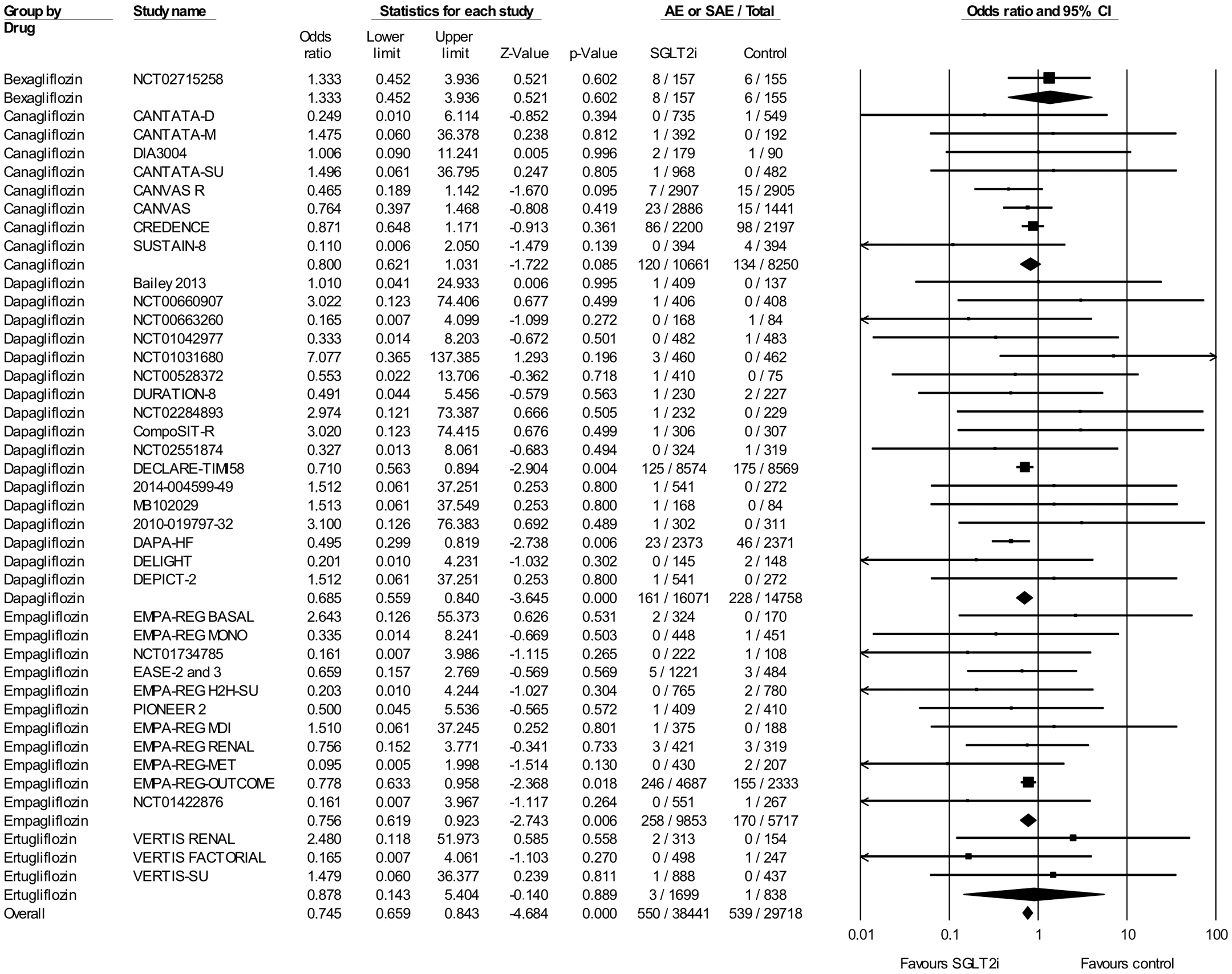

Supplement: S4 Fig — AKI, acute kidney injury; RCT, randomized controlled trial; SGLT2i, sodium-glucose cotransporter-2 inhibitor. (TIF) [file pmed.1002983.s010.tif]

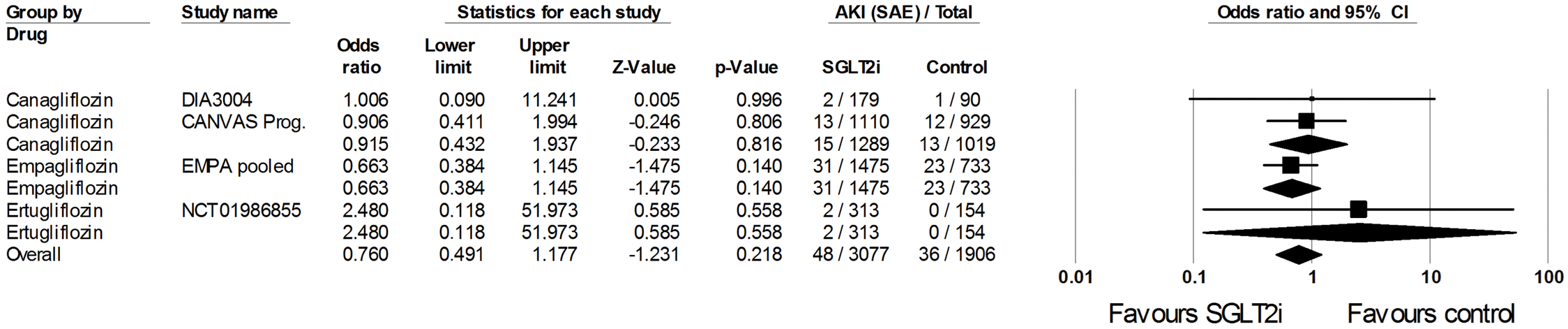

Supplement: S5 Fig — AE, adverse event; AKI, acute kidney injury; eGFR, estimated Glomerular Filtration Rate; SAE, serious AE; SGLT2i, sodium-glucose cotransporter-2 inhibitor. (TIF) [file pmed.1002983.s011.tif]

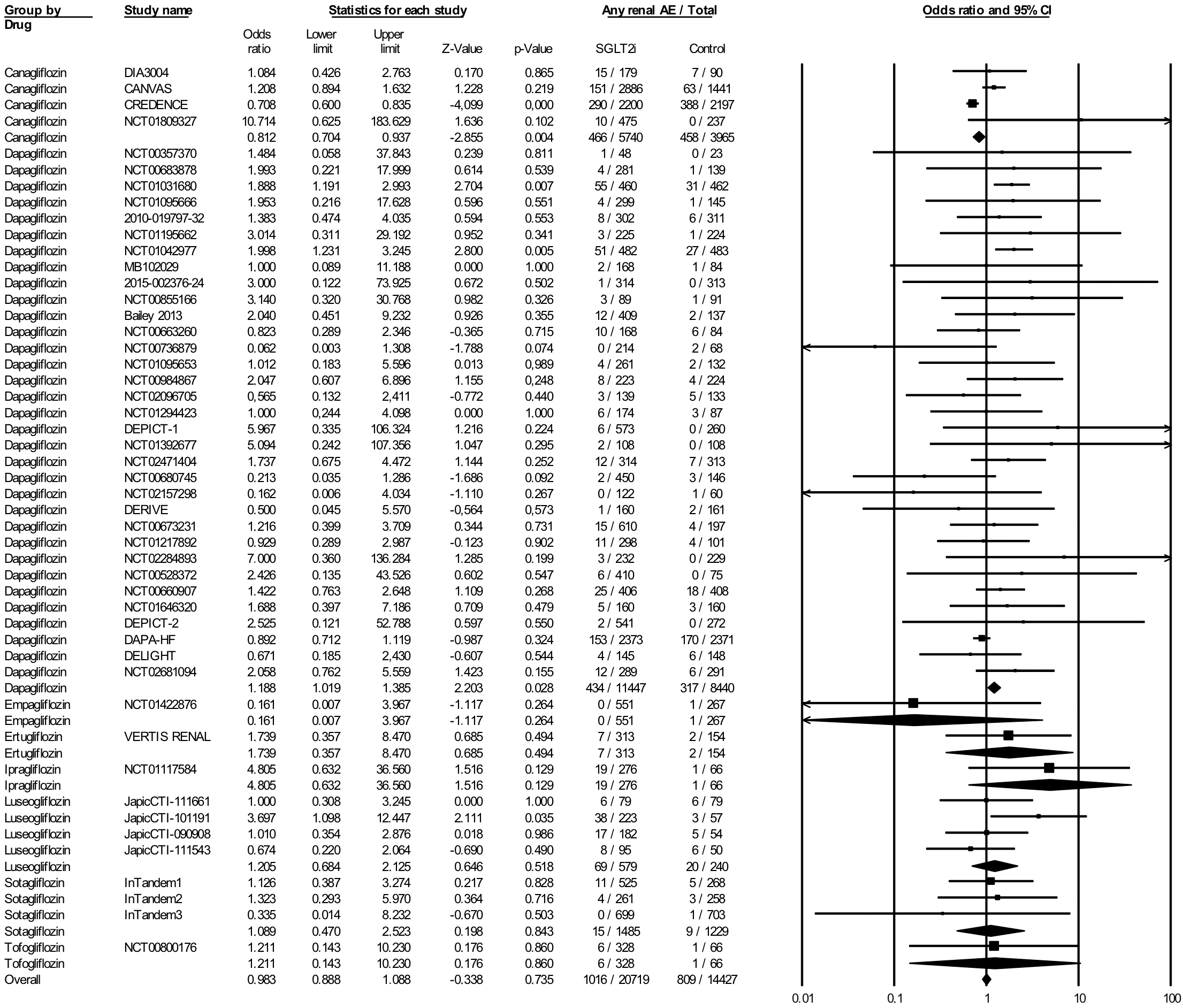

Supplement: S6 Fig — AE, adverse event; RCT, randomized controlled trial; SGLT2i, sodium-glucose cotransporter-2 inhibitor. (TIF) [file pmed.1002983.s012.tif]

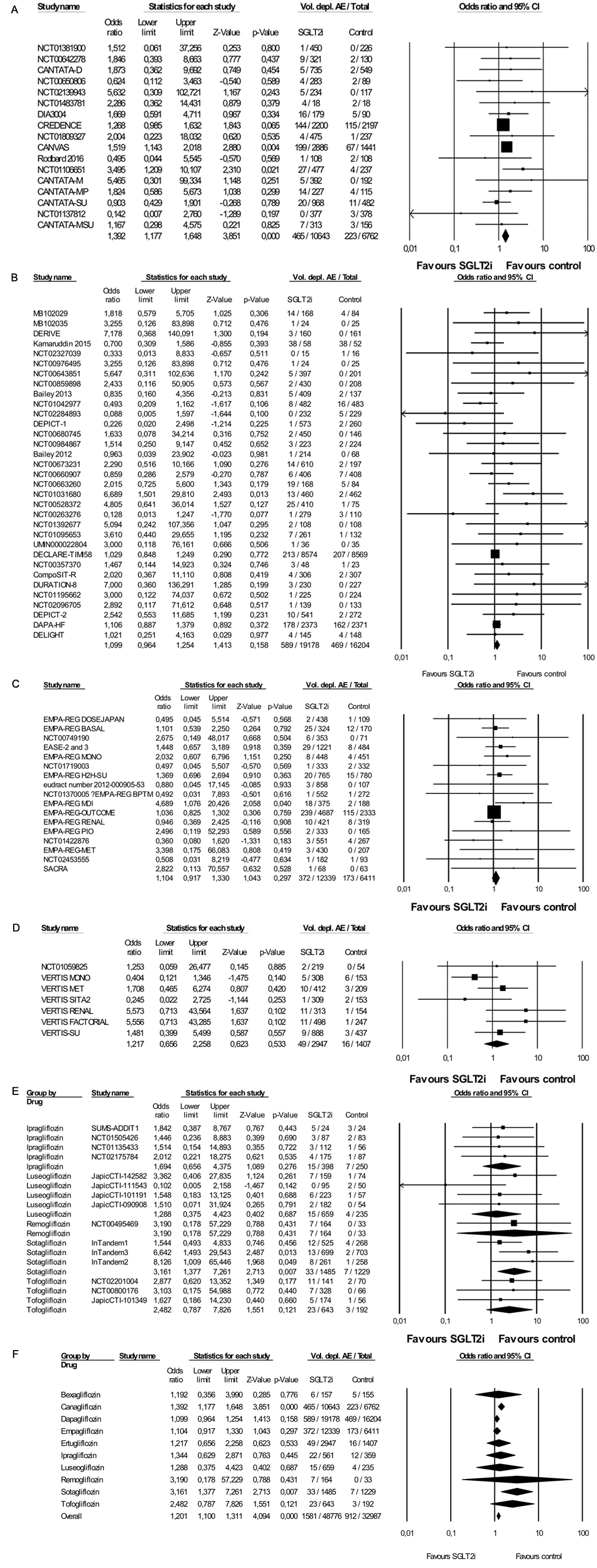

Supplement: S7 Fig — (a) Canagliflozin, (b) dapagliflozin, (c) empagliflozin, (d) ertugliflozin, (e) other SGLT2is, and (f) comparison of estimates of all examined drugs. AE, adverse event; RCT, randomized controlled trial; SGLT2i, sodium-glucose cotransporter-2 inhibitor. (TIF) [file pmed.1002983.s013.tif]

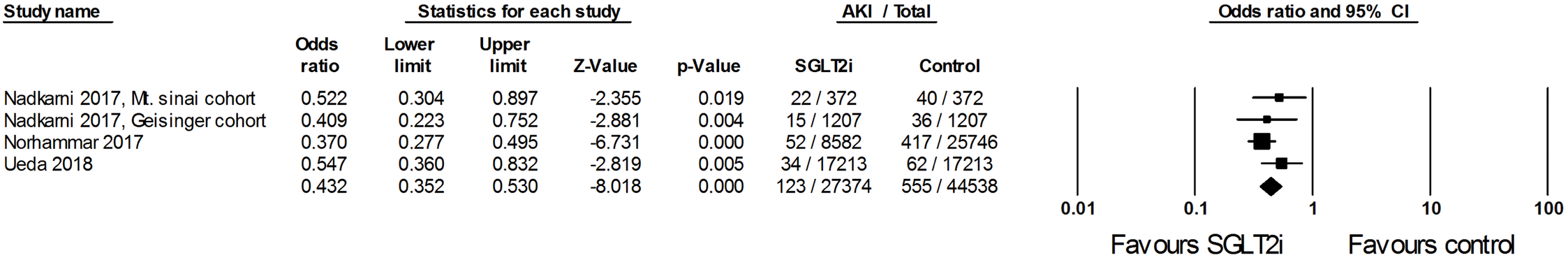

Supplement: S8 Fig — AKI, acute kidney injury; SGLT2i, sodium-glucose cotransporter-2 inhibitor. (TIF) [file pmed.1002983.s014.tif]
