# Supplementary material for: Acute kidney injury and adverse renal events in patients receiving SGLT2-inhibitors: A systematic review and meta-analysis
Source: PLoS Med. 2019 Dec 9;16(12):e1002983. doi: 10.1371/journal.pmed.1002983 (PMC6901179; doi:10.1371/journal.pmed.1002983)
Supplement: Attachment — Submitted filename: Revision_Responses_3.10.19.docx [file pmed.1002983.s015.docx]

Requests from the editors:

Abstract – please avoid the list like appearance and sectioning. The abstract should have 3 sections: Background, Methods and Findings and Conclusions. The final sentence of the Methods and Findings should be the limitations of the study. Please look at other published systematic reviews to see formatting for an abstract.

***We thank the editors for pointing this out; we have rearranged the abstract accordingly (page 2).***

At this stage, we ask that you include a short, non-technical Author Summary of your research to make findings accessible to a wide audience that includes both scientists and non-scientists. The Author Summary should immediately follow the Abstract in your revised manuscript. This text is subject to editorial change and should be distinct from the scientific abstract. Please see our author guidelines for more information: <https://journals.plos.org/plosmedicine/s/revising-your-manuscript#loc-author-summary>

***We have included a summary paragraph in non-technical language (page 3).***

Please use square brackets in the main text for references numbers instead of superscript.

***We have amended the referencing format accordingly throughout.***

Study Selection (Two independent investigators reviewed all articles) – please give initials of these authors. And then those for the 3rd.

***We have made the requested changes (page 4).***

Remove “Role of the Funding Source: No external funding.” From the main text

***We have removed this paragraph.***

I note some use of causal language (eg from abstract “SGLT2i prevents AKI with”). Even though this SR does feature trials there is also the inclusion of observational studies. Please avoid overheated language.

***We agree with the editors’ comments and have amended the respective paragraphs (Abstract, page 8).***

Page 6 – “that there is indeed some tubular alteration” may instead of is.

***We have amended the sentence accordingly (page 8)***

Insert a subheading ‘Conclusions’ page 9

***We have inserted a corresponding paragraph.***

Remove conflict of interest from the main text.

***We have removed this paragraph.***

**Comments from the reviewers:**

Reviewer #1: Nice review, up-to-date on methods. Data are in line with results from the major large CV and renal outcome studies.

- Suggest to edit figures because the point estimated of the smaller studies are not visible.

***We have redrawn the figures so the point estimates are more readily observable by increasing the symbols of the point estimates. As the symbols are proportional this is limited by the size of the larger studies getting very big.***

- suggest to analyse a subgroup of studies that were double-blind

***Our analysis overall included only seven studies that were not double-blind. To show the effect of removing them we repeated the analysis for our primary endpoint (SAE-AKI) by removing the one study that had contributed to this endpoint. The OR did not change significantly (0.646 in this analysis vs. 0.642 in the full analysis set).***

- suggest to explain in the discussion that most randomised patients in the large trials received inhibitor of the RAS. Thus post-glomerular dilation (RASi) combined with pre-glomerular constriction (SGLT2i) should increase risk of AKI. The opposite was found and there is animal studies of experimental ischemic AKI showing much the same.

***We thank the reviewer for the helpful comments. In response, we have:***

- ***Included a table to show the number of patients on RAS inhibitors in those trials were the corresponding data was available (Table S4, see page 36).***
- ***We have also added a discussion of the pre- and post-glomerular dilatation effects of combined SGLT2 and RAS inhibition in the discussion (page 8).***

Reviewer #2: Alex McConnachie

Menne et al report a meta-analysis of RCTs and observational studies of the effect of SGLT-2 inhibitors on AKI. This review looks at the use of statistics in the paper.

I have no major concerns with the analyses presented in the paper, just a few minor observations.

First, a typo: the final paragraph refers to the use of a "Funnel blot".

***We have corrected the spelling (page 5).***

Then, an issue with wording: the second paragraph of the results states that "SGLT2i diminished the odds ratio by 32%"; this should perhaps be "SGLT2i diminished the odds by 32%".

***We agree with the editor and have amended the wording accordingly (page 5).***

There are a few places in the paper where the authors make statements about the effects of different SGLT-2 inhibitors being similar (e.g. in the section headed "AEs of AKI in RCT") or different (under "Hypovolemia related AE"), though they do not present any statistical tests to back up these statements. For hypovolemia, in particular, some sort of interaction test would be useful.

***We thank the editor for pointing this out. When talking about comparisons between drugs we have now included the respective p-values of the comparisons (page 6).***

When talking about AKI in observational studies, there is a little confusion as to whether there are four studies, or five.

***The confusion arises because two separate observational cohorts have been published jointly in the paper by Nadkarni et al, resulting in 5 cohort studies being reported in 4 publications (see Table S3). We have included a clarification statement on page 7.***

In the first paragraph of the discussion, it is stated that SLGT-2i reduce the incidence of acute renal failure. Is "incidence" correct here, or should "odds" be used?

***We agree the correct term would be reduced odds, and have amended the sentence accordingly.***

Given that the included studies will have different lengths of follow-up, to what extent are the odds ratios directly comparable between studies? This could perhaps be listed among the limitations of the analysis.

***We agree that this represents a limitation of the analysis, and we have added the varied follow-up duration to our list of limitations in the discussion (page 8). To increase data transparency we have listed the duration of each study in summary Table S2.***

The figures are generally good, though S8 is extremely hard to read. Would it be better to break it up into a number of figures, perhaps one for each individual drug, then give one showing how the drug-specific estimates combine to give an overall SGLT-2i effect estimate?

***We agree and have broken down figure 8 into separate figures S8a - f, as well as a summary figure of drug-specific estimates.***

Reviewer #3: Well written, timely and clinically important. Although the emphasis was on AKI, I think it is also important to include all genitourinary AEs such as UTIs in this report.

***We thank the reviewer for the encouraging comment. We agree the issue of genitourinary side effects is pertinent to SGLT2 inhibitors as it influences prescribing decisions. However, several well-conducted systematic reviews on the topic of AEs, including infectious complications, have already been published and shown an increased risk of genital infections, but not urinary infections (see e.g. Puckrin et al 2018*** [***https://www.ncbi.nlm.nih.gov/pubmed/29484489***](https://www.ncbi.nlm.nih.gov/pubmed/29484489)***, Li et al 2017*** [***https://www.ncbi.nlm.nih.gov/pubmed/27862830***](https://www.ncbi.nlm.nih.gov/pubmed/27862830)***, or Yang et al 2019*** [***https://www.ncbi.nlm.nih.gov/pubmed/31376072***](https://www.ncbi.nlm.nih.gov/pubmed/31376072)***), and there is currently no evidence to suggest our main outcome of interest (AKI) is related to infectious complications (as opposed to e.g. volume depletion), so we feel a separate analysis of this AE would contribute little to the current state of knowledge and reduce the focus and clarity of our analysis.***

Reviewer #4: In the systematic review, the authors have included randomized control trials as well as observational studies of an SGLT2 inhibitor compared to controls. The outcomes were acute kidney injury, as defined on the basis of serious adverse event or adverse event reporting for the trials. In addition they also report hypovolemia similarly defined from the trials. Lastly they also have observational studies which reported acute kidney injury events.

Overall the authors report that the risk of acute kidney injury is decreased by about 24 to 32%, depending on which definition is used, and the trials and by 60% in the observational studies. In contrast, hypovolemia is increased by about 20%.

Overall the results do seem valid, however, this reviewer has some concerns about the interpretation, especially given the high heterogeneity, with respect to the difference between agents. The patient population and the studies does vary quite a lot, with the population in CREDENCE being at higher risk than in the other large trials. This may cast canagliflozin and an unfavorable light in some analysis and in favorable light in some other analysis. It is unlikely that these are true differences, versus a difference in baseline the risk and these conclusions should be drawn very cautiously.

***We thank the reviewer for pointing this out. At various places in the manuscript we had erroneously quoted the number of the Q value instead of I^2^. This made a significant change to our interpretation of the degree of heterogeneity only in the case of “volume depletion”, for whom the first version of the manuscript erroneously stated high heterogeneity had been observed, when in fact there was no real heterogeneity (I^2^ <0.001). Comparison of effects across the different drugs showed no statistically significant difference (p=0.26). We observed considerable heterogeneity only in the composite renal AE endpoint (in which canagliflozin did not perform worse than the other drugs, with an OR for combined renal AEs of 0.81, CI 0.70-0.94)). For all other endpoints the heterogeneity was 0. These mistakes have been corrected in the text on page 6 and 7, and we apologise for causing confusion. Furthermore we changed the section about volume depletion in the discussion (Page 8).***

The other major concern is that most of the data in this systematic review comes from the for major trials, CANVAS, EMPAREG, CREDENCE and DECLARE. The remaining trials add a very few numbers in the overall analysis. Unfortunately for the authors, a competing systematic review has recently been published which also reports a similar findings on acute kidney injury (25% RRR)from these 4 trials combined together <https://www.thelancet.com/journals/landia/article/PIIS2213-8587(19)30256-6/fulltext>. The difference of the present manuscript, is that they include different definitions of acute kidney injury, observational studies, and the outcome of hypovolemia. Separating the trial outcomes [ AKI and hypovolemia] and the observational studies [ acute kidney injury] would be helpful in the flow of the manuscript to make this apparent.

***We agree that some of the information presented in our paper is also included in the recently published review by Neuen et al, but feel that with our less restrictive inclusion criteria, with consideration of smaller trials and observational cohorts in a real-world setting, we present a fuller analysis of the evidence regarding AKI risk. We have rearranged the results section to move the “AKI in observational studies” paragraph to the bottom of page 6 and amended the wording to again emphasise that this constituted a separate aspect of our analysis.***

***Furthermore, we have re-run our database search to also cover the period from 04/04/19 – 27/09/19. This yielded 8 further studies for inclusion, including the recently published large DAPA-HF trial which also reported on AKI outcomes and is not included in the review by Neuen et al. The additional studies strengthened our conclusions (s. amended figures throughout).***

Though this was not prespecified, some more analyses could help this paper stand out even more. For example, in the analysis where high heterogeneity was reported, some exploration of the heterogeneity would be useful. This could be done either a subgroup analysis or as Metaregressions. Understandably, these would all be post hoc analysis, however they may shed some more light on the underlying statistical heterogeneity.

***As pointed out before, we could only detect heterogeneity for the endpoint “any renal event”. For this endpoint we performed some additional analyses. First we did a subgroup analysis of the different drugs used. In a mixed effects analysis, this did not reveal a difference between drugs (p=0.722).***

***In addition we also explored the importance of HbA1c, eGFR and BP by metaregression analysis showing that higher HbA1c, lower eGFR and higher BP are associated with a favourable effect of SGLT2 inhibition.***

In addition we also explored the importance of eGFR, HbA1c and BP on the “volume” endpoint. Here we did not see any influence:

Otherwise the discussion is clearly written, and emphasizes the lack of a mechanistic explanation, as well as the next steps.

***We thank the reviewer for the helpful comments.***
