# Supplementary material for: Acute kidney injury and adverse renal events in patients receiving SGLT2-inhibitors: A systematic review and meta-analysis
Source: PLoS Med. 2019 Dec 9;16(12):e1002983. doi: 10.1371/journal.pmed.1002983 (PMC6901179; doi:10.1371/journal.pmed.1002983)
Supplement: Attachment — Submitted filename: Revisions.docx [file pmed.1002983.s016.docx]

Reformatting

- Figures 3, and 4 appear to contain decimals marked with commas instead of decimal points (i.e., "0,05" instead of "0.05"). Please punctuate decimal numbers with decimal points.

**We have removed the remaining commas and apologize for this oversight.**

- You have included reference citations in your S2 and S3 Tables. Since each Supporting Information file is self-contained and cannot link back to the main manuscript where you've listed those references, we ask that you please list all references cited in S2 Table and S3 Table in full in each specific file under a 'References' heading, and begin the numbering the citations at 1 in each supporting information file. Any references in the main manuscript's reference list that are not cited in the main manuscript should be removed. Please renumber the main manuscript references and citations if needed so that they remain in ascending numerical order.

**We have added the references to the supporting information files and changed the numbering in the supporting information tables and the main manuscript.**
